# Supplementary figures and images for: Geospatial distribution of Hepatitis E seroprevalence in Nepal, 2021
Source: PLoS Negl Trop Dis. 2024 Dec 23;18(12):e0012746. doi: 10.1371/journal.pntd.0012746 (PMC11706507; doi:10.1371/journal.pntd.0012746)

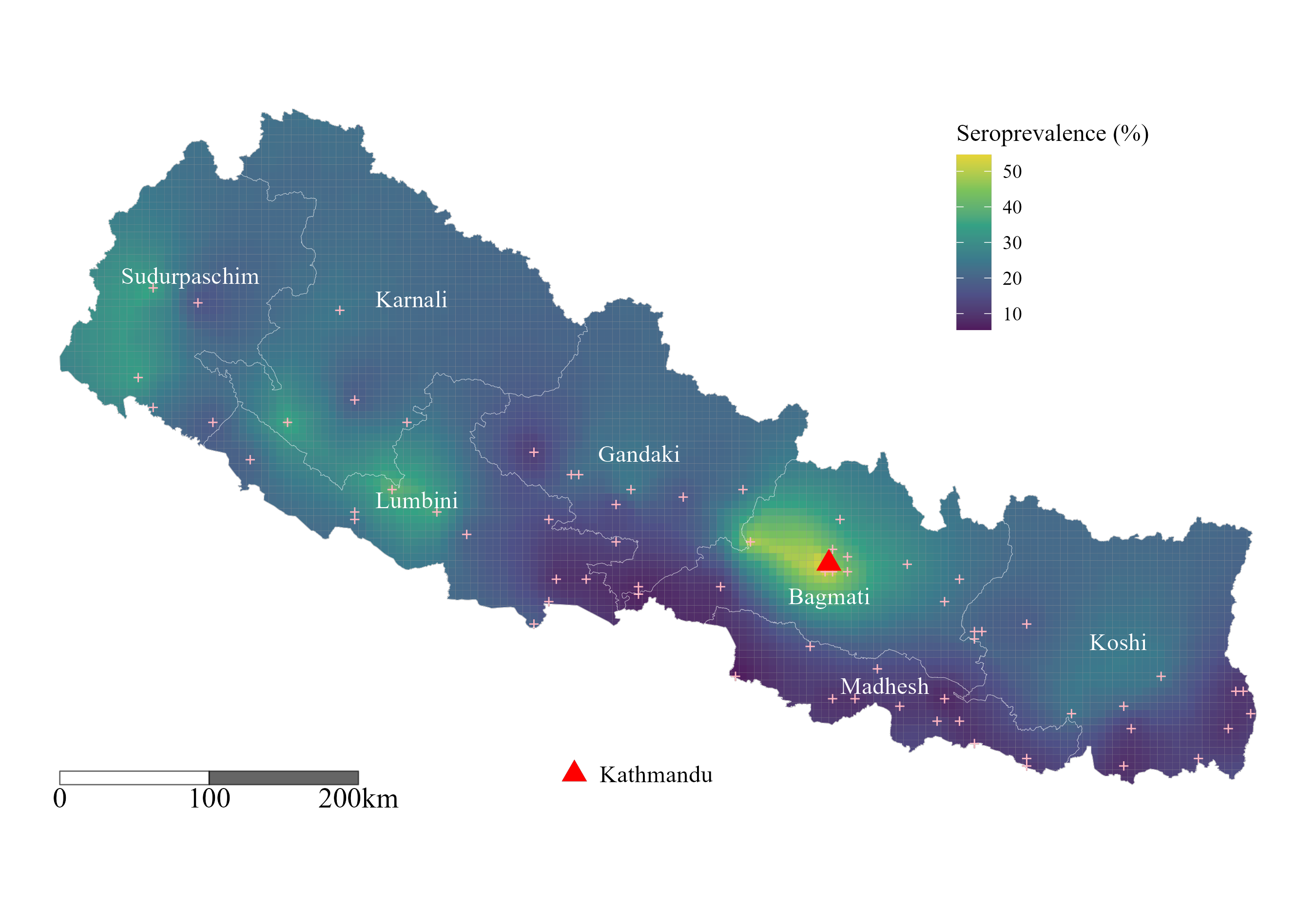

Supplement: S1 Fig — Predicted percentage of people with antibodies to HEV from the best fitting geostatistical model (the null model with spatial random effects only). Sampled locations are indicated by pink crosses and Kathmandu is marked with a red triangle. The map was produced in R version 4.2.1 (https://www.R-project.org/). The boundaries of the seven provinces of Nepal were made using the open-source shapefiles and spatial data made available under a Creative Commons Attribution for Intergovernmental Organisations license from the Humanitarian Data Exchange [17]. Both the open-source files and the licensing information are available at this direct link: https://data.humdata.org/dataset/cod-ab-npl. (TIF) [file pntd.0012746.s001.tif]
