# Supplementary material for: Geospatial distribution of Hepatitis E seroprevalence in Nepal, 2021
Source: PLoS Negl Trop Dis. 2024 Dec 23;18(12):e0012746. doi: 10.1371/journal.pntd.0012746 (PMC11706507; doi:10.1371/journal.pntd.0012746)
Supplement: Attachment — Submitted filename: comments.docx [file pntd.0012746.s002.docx]

# Peer Review for Manuscript: Seroprevalence and Geospatial Distribution of Hepatitis E Seropositivity in Nepal, 2021

Below are specific suggestions on areas needing improvement to enhance the manuscript’s clarity, context, and impact.

## Abstract:

**Lines 16-22:** Ensure consistency in terminology, such as “seroprevalence” and “seropositivity.”

- Current: “We analyzed existing samples from a nationally representative serosurvey to describe the geospatial distribution and factors associated with HEV seropositivity in Nepal, as a proxy for infection.”
- Suggested: “We analyzed existing samples from a nationally representative serosurvey to describe the geospatial distribution and factors associated with HEV seroprevalence in Nepal, as a proxy for infection.”

**Line 31:** Rephrase for clarity.

- Current: “Seroprevalence increased with age, and was higher in males…”
- Suggested: “HEV seroprevalence increased with age and was higher in males…”

## Introduction

**Lines 55-74:** Provide a brief overview of previous HEV studies in Nepal to enhance context. Mention any existing data or studies that have explored HEV prevalence in Nepal to set the stage for the novelty and necessity of your research.

- Suggested addition: “Previous studies on HEV in Nepal have primarily focused on outbreak investigations and small-scale surveys, leaving a gap in comprehensive national-level data. Notably, Shrestha et al.
  (2006) reported HEV as responsible for 50% of acute hepatitis cases in Kathmandu, highlighting the virus’s significant impact in urban areas.”

**Lines 58-59:** The sentence could be more specific about the impact of HEV.

- Suggested: “Hepatitis E virus (HEV) causes acute jaundice, particularly impacting liver function, and poses a significant public health problem in low- and middle-income countries.”

**Lines 64-66:** Clarify the point about underreporting.

- Current: “Limited surveillance capacity and suboptimal access to diagnostics mean that the burden of HEV infections in affected countries, including Nepal, is likely to be severely underreported.”
- Suggested: “Due to limited surveillance capacity and suboptimal access to diagnostics, the true burden of HEV infections in countries like Nepal is likely underreported.”

## Methods

### Survey Design:

- **Lines 85-103:** Clarify the criteria for selecting wards and households more explicitly. Explain if there were any specific characteristics considered in the selection process beyond population size.
  - Suggested addition: “Wards were selected based on population size and geographic diversity to ensure representativeness across different ecological regions and municipality types. Households within these wards were then chosen using systematic random sampling, taking into account factors such as household density and accessibility.”

### Laboratory Analysis:

- **Lines 104-110:** Include details about the sensitivity and specificity of the Wantai immunoassay used for testing anti-HEV IgG. Explain why this particular assay was chosen over others.
  - Suggested addition: “The Wantai immunoassay, known for its high sensitivity (99%) and specificity (98%), was chosen based on its robust performance in previous HEV studies.”
- **Lines 107-109:** Clarify the handling of borderline samples.
  - Current: “Borderline samples were retested, and consistently borderline samples were excluded from the analysis.”
  - Suggested: “Samples with optical density values close to the cutoff (0.9-1.1) were retested, and those consistently remaining borderline were excluded from the final analysis to ensure accuracy.”

### Variable Definition:

- **Lines 112-123:** Elaborate on the wealth index calculation for better reproducibility. Include a brief description of the eight questions used and how responses were weighted.
  - Suggested addition: “The wealth index was derived from responses to eight questions related to household assets and living conditions, such as ownership of durable goods, housing materials, and access to utilities. Each item was assigned a weight based on its importance in the context of socioeconomic status, following the methodology of the Nepal Demographic Health Survey 2016.”
- **Lines 115-118:** Rephrase for clarity.
  - Current: “Sources of water and household toilet facility were categorized as improved and unimproved according to the World Health Organization/United Nations Children’s Fund Joint Monitoring Programme for Water Supply, Sanitation and Hygiene criteria.”
  - Suggested: “Sources of water and household toilet facilities were categorized as ‘improved’ or ‘unimproved’ based on the criteria set by the World Health Organization and the United Nations Children’s Fund Joint Monitoring Programme for Water Supply, Sanitation, and Hygiene.”

## Statistical Analysis:

- **Lines 125-143:** Provide a justification for using hierarchical logistic spatial regression models and discuss any assumptions made. Explain why Bayesian models were chosen and the advantages they offer in this context.
  - Suggested addition: “Hierarchical logistic spatial regression models were employed to account for the multilevel structure of the data, capturing both individual-level and area-level variations in HEV seroprevalence. Bayesian methods were chosen for their ability to incorporate prior information and provide robust estimates even with complex spatial dependencies. This approach also allows for more accurate uncertainty quantification in the presence of spatial autocorrelation.”

## Results

- **Lines 145-177:** Highlight only key statistical results (e.g., p-values) in the text for important comparisons. Explicitly state the statistical significance of differences in seroprevalence between regions.
  - Suggested addition: “The differences in seroprevalence between the hilly (28.9%, 95% CI: 26.6-31.2) and terai (14.2%, 95% CI: 12.7-15.8) regions were statistically significant (p < 0.001), indicating a higher burden of HEV in the hilly regions.”

### Tables and Figures:

- Ensure all figures are high resolution and clearly labeled. Consider adding more descriptive captions that summarize the key takeaways from each figure/table.
  - Suggested addition for Table 1 caption: “Table 1. Demographic characteristics of study participants and HEV seroprevalence by demographic factors.”
  - Suggested addition for Figure 1 caption: “Figure 1. Hepatitis E Seroprevalence and 95% Confidence Intervals across different demographic and geographic groups.”

## Discussion

- **Lines 179-247:** Discuss potential policy implications and recommendations for future research more explicitly. Identify specific actions that could be taken based on the study’s findings, such as targeted interventions in high-prevalence areas.
  - Suggested addition: “Our findings suggest the need for targeted interventions in regions with high seroprevalence, particularly the hilly and mountain areas. Public health initiatives should focus on improving water quality and sanitation in these regions. Additionally, the implementation of HEV vaccination programs in high-risk populations, including pregnant women and individuals with chronic liver disease, could significantly reduce the disease burden. Future research should explore the role of zoonotic transmission and the potential benefits of expanding HEV vaccination to broader age groups.”
- **Lines 201-203:** Discuss the limitations in more depth.
  - Current: “The seroprevalence of HEV IgG as a surrogate marker for past infection is likely to be an underestimate as antibodies against HEV are known to wane over time.”
  - Suggested: “The seroprevalence of HEV IgG as a surrogate marker for past infection may underestimate the true infection rate, as antibodies against HEV are known to wane over time. This decline in antibody levels, known as seroreversion, could lead to false negatives, particularly among those infected in the distant past. Additionally, the cross-sectional nature of the study limits the ability to infer causal relationships.”

## Conclusion

- **Lines 249-255:** Reinforce the importance of the study’s findings for public health planning and resource allocation. Suggest specific areas for intervention based on the geographic distribution of HEV seropositivity.
  - Suggested addition: “The high seroprevalence in Kathmandu and other urban areas underscores the urgent need for targeted surveillance and intervention programs. Strengthening water, sanitation, and hygiene (WASH) initiatives and considering HEV vaccination for high-risk groups are crucial steps for reducing the burden of HEV in Nepal.”

## Overall Assessment

The manuscript is well-structured and provides significant insights into HEV seroprevalence in Nepal. Addressing the suggestions mentioned above will further enhance the manuscript’s clarity, context, and impact. The detailed proofreading notes aim to improve readability and precision.

## Recommendations

1. **Minor Revision:** Address the suggestions and proofreading notes provided above.
2. **Ethical Approval:** Ensure all ethical considerations are clearly documented, particularly regarding bio-banking and informed consent.
3. **Future Research:** Include a brief discussion on future research directions and policy implications based on the findings. Provide more detailed recommendations for policymakers and public health practitioners.

## Specific Areas for Improvement

1. **Introduction:** Provide more background on HEV epidemiology in Nepal and previous studies to set the stage for the current research.
2. **Methods:** Clarify sampling criteria, provide more detail on laboratory quality control, and justify the choice of statistical models.
3. **Results:** Highlight key statistical findings more clearly and discuss observed patterns in greater detail.
4. **Discussion:** Expand on policy implications and future research directions. Address limitations in more depth.
5. **Figures and Tables:** Ensure high-resolution images and add more descriptive captions. Consider including additional visualizations to illustrate regional differences.
6. **References:** Update your references to the most recent literature, the oldest shoud be five years ago and not beyond that, also ensure consistency in formatting. Include more recent studies.
