# Supplementary material for: Geospatial distribution of Hepatitis E seroprevalence in Nepal, 2021
Source: PLoS Negl Trop Dis. 2024 Dec 23;18(12):e0012746. doi: 10.1371/journal.pntd.0012746 (PMC11706507; doi:10.1371/journal.pntd.0012746)
Supplement: Attachment — Submitted filename: Response to Reviewers_10OCT2024.docx [file pntd.0012746.s003.docx]

Oct 10, 2024

Dear Reviewers,

**Title: Seroprevalence and Geospatial Distribution of Hepatitis E Seropositivity in Nepal, 2021(PNTD-D-24-00751)**

Thank you very much for your detailed review and helpful suggestions. We really appreciate it and feel it has improved the clarity of our manuscript. On the behalf of my co-authors, I am submitting the enclosed manuscript entitled [Seroprevalence and Geospatial Distribution of Hepatitis E Seropositivity in Nepal, 2021(PNTD-D-24-00751)] with point-by-point responses to the comments from the reviewers.

## Abstract:

**Lines 16-22:** Ensure consistency in terminology, such as “seroprevalence” and “seropositivity.”

- Current: “We analyzed existing samples from a nationally representative serosurvey to describe the geospatial distribution and factors associated with HEV seropositivity in Nepal, as a proxy for infection.”
- Suggested: “We analyzed existing samples from a nationally representative serosurvey to describe the geospatial distribution and factors associated with HEV seroprevalence in Nepal, as a proxy for infection.”

**Author Response**: We have revised the manuscript by using “seroprevalence” throughout the paper for consistency. Per reviewer’s suggestion, we have revised the Background section of the abstract as below.

**Line 20-22:** **We analyzed existing samples from a nationally representative serosurvey to describe the geospatial distribution and factors associated with HEV seroprevalence in Nepal, as a proxy for infection**.

In addition, to be consistent throughout the journal, we have modified the title of the journal as [**Geospatial Distribution of Hepatitis E Seroprevalence in Nepal, 2021**].

**Line 31:** Rephrase for clarity.

- Current: “Seroprevalence increased with age, and was higher in males…”
- Suggested: “HEV seroprevalence increased with age and was higher in males…”

**Author Response**: We revised per reviewer’s suggestion as below.

**Line31-32:** **HEV seroprevalence increased with age, and was higher in males (23.5%, 95% CI: 21.5 – 25.5) than in females (18.6%, 95% CI: 16.9 – 20.3).**

## Introduction

**Lines 55-74:** Provide a brief overview of previous HEV studies in Nepal to enhance context. Mention any existing data or studies that have explored HEV prevalence in Nepal to set the stage for the novelty and necessity of your research.

- Suggested addition: “Previous studies on HEV in Nepal have primarily focused on outbreak investigations and small-scale surveys, leaving a gap in comprehensive national-level data. Notably, Shrestha et al. reported HEV as responsible for 50% of acute hepatitis cases in Kathmandu, highlighting the virus’s significant impact in urban areas.”

**Author Response**: Following reviewer’s suggestion, we have revised phrases on the Introduction section of the main body as below. After providing a brief overview of previous HEV studies in Nepal in the second paragraph, we have highlighted limitations of the existing literature and why we aimed to conduct nationally representative serosurvey for HEV seroprevalence.

**Line 77-85**: Despite several studies reporting endemicity of HEV in Nepal, nationally representative disease burden data on HEV are lacking. **Previous studies on HEV in Nepal have primarily focused on outbreak investigations and small-scale surveys, leaving a gap in comprehensive national-level data**. **Population-based HEV epidemiologic data are needed to support informed decision making on strengthening the national waterborne disease surveillance system and to generate a comprehensive control plan. In the absence of consistent case-based surveillance data, seroprevalence can provide an important proxy measure of infection.** Therefore, we conducted a study to estimate HEV seroprevalence and describe the geospatial distribution using banked samples from a previously conducted cross-sectional sero-survey for estimating burden of cholera in Nepal.

**Lines 58-59:** The sentence could be more specific about the impact of HEV.

- Suggested: “Hepatitis E virus (HEV) causes acute jaundice, particularly impacting liver function, and poses a significant public health problem in low- and middle-income countries.”

**Author Response:** Following reviewer’s suggestion, we have revised the first paragraph of the Introduction as below. We have described burden of HEV briefly and why it poses a significant public health problem in LMICs.

**Line 57:63**: **Hepatitis E virus (HEV) causes acute jaundice by impacting liver function. HEV poses a significant public health problem in many low- and middle-income countries (LMICs) where genotypes 1 and 2 are endemic, spreading from person-to-person via contaminated water** [1]. Despite causing large outbreaks of acute jaundice and being associated with a high case fatality rate among pregnant women [2], HEV is often considered a neglected disease as the majority of HEV infections remain undetected due to self-limiting clinical characteristics, poor surveillance, and limited access to healthcare [3]. Thus, global burden estimates are highly variable.

**Lines 64-66:** Clarify the point about underreporting.

- Current: “Limited surveillance capacity and suboptimal access to diagnostics mean that the burden of HEV infections in affected countries, including Nepal, is likely to be severely underreported.”
- Suggested: “Due to limited surveillance capacity and suboptimal access to diagnostics, the true burden of HEV infections in countries like Nepal is likely underreported.”

**Author Response**: Following reviewer’s suggestion, we have revised the first paragraph of the Introduction as below, clarifying the point about under-reporting of HEV infections.

**Line 63:67**: A systematic review conducted in 2020 estimated approximately 939 million people, corresponding to 1 in 8 individuals, have experienced HEV infection globally and 15‐110 million individuals have recent or ongoing HEV infection [4]. **Due to limited surveillance capacity and suboptimal access to diagnostics, the true burden of HEV infections in countries like Nepal is likely underreported.**

## Methods

### Survey Design:

- **Lines 85-103:** Clarify the criteria for selecting wards and households more explicitly. Explain if there were any specific characteristics considered in the selection process beyond population size.
  - Suggested addition: “Wards were selected based on population size and geographic diversity to ensure representativeness across different ecological regions and municipality types. Households within these wards were then chosen using systematic random sampling, taking into account factors such as household density and accessibility.”

**Author Response**: We have provided additional details on how Nepal is divided administratively and ecologically. Then we added more details on sampling method per reviewer suggestion. To better clarify the process, we have inserted Figure 1 to briefly summarize sampling method and consort diagram of study participants.

**Line 91-101**: **Nepal is divided into seven provinces: Koshi, Madhesh, Bagmati, Gandaki, Lumbini, Karnali, and Sudurpashchim. Provinces are divided into districts, districts into municipalities, and municipalities into wards. Each ward can be categorized as either urban or rural depending on its administrative municipality type. The number of households in rural wards typically range between 150-300, while wards in urban area can go up to 800 or more. Apart from administrative classification, Nepal has three distinctive ecological regions (terai, hilly, or mountain). To ensure national representativeness, two-step cluster random sampling was performed. First, 65 wards (primary sampling unit) were selected based on population size and geographic diversity to ensure representativeness across different ecological regions and municipality types.** Then 15 households were selected per ward using systematic random sampling after household enumeration, taking into account factors such as household density and accessibility.

### Laboratory Analysis:

- **Lines 104-110:** Include details about the sensitivity and specificity of the Wantai immunoassay used for testing anti-HEV IgG. Explain why this particular assay was chosen over others.
  - Suggested addition: “The Wantai immunoassay, known for its high sensitivity (99%) and specificity (98%), was chosen based on its robust performance in previous HEV studies.”
- **Lines 107-109:** Clarify the handling of borderline samples.
  - Current: “Borderline samples were retested, and consistently borderline samples were excluded from the analysis.”
  - Suggested: “Samples with optical density values close to the cutoff (0.9-1.1) were retested, and those consistently remaining borderline were excluded from the final analysis to ensure accuracy.”

**Author Response**: Per reviewer suggestions, we have added additional detail about laboratory kits used for the study including sensitivity and specificity, and how the borderline samples were handled for analysis.

**Line 114-120**: Available banked sera from participants who consented to long-term storage and future analysis were tested for anti-HEV Immunoglobulin G (IgG) using the commercially available **Wantai immunoassay (Beijing Wantai Biological Pharmacy Enterprise Co Ltd.) per manufacturer’s instruction at Dhulikhel Hospital, Nepal. The Wantai immunoassay, known for its high sensitivity (99%) and specificity (98%), was chosen based on its robust performance in previous HEV studies** [9, 10]. Samples with standardized optical density ≥ 1.0 were interpreted as positive, < 1 as negative, and 0.9-1.1 as borderline. **Borderline samples were retested, and consistently borderline samples were excluded from the analysis**.

### Variable Definition:

- **Lines 112-123:** Elaborate on the wealth index calculation for better reproducibility. Include a brief description of the eight questions used and how responses were weighted.
  - Suggested addition: “The wealth index was derived from responses to eight questions related to household assets and living conditions, such as ownership of durable goods, housing materials, and access to utilities. Each item was assigned a weight based on its importance in the context of socioeconomic status, following the methodology of the Nepal Demographic Health Survey 2016.”
- **Lines 115-118:** Rephrase for clarity.
  - Current: “Sources of water and household toilet facility were categorized as improved and unimproved according to the World Health Organization/United Nations Children’s Fund Joint Monitoring Programme for Water Supply, Sanitation and Hygiene criteria.”
  - Suggested: “Sources of water and household toilet facilities were categorized as ‘improved’ or ‘unimproved’ based on the criteria set by the World Health Organization and the United Nations Children’s Fund Joint Monitoring Programme for Water Supply, Sanitation, and Hygiene.”

**Author Response**: We have elaborated additionally on previously validated simplified wealth index and rephrased how sources of water and toilet facility were categorized.

**Line 123-136**: The classification of province, municipality type, and ecological region adhered to Nepal's administrative guidelines. **Sources of water and household toilet facilities were categorized as ‘improved’ or ‘unimproved’ based on the criteria set by the World Health Organization and the United Nations Children’s Fund Joint Monitoring Programme for Water Supply, Sanitation, and Hygiene** [11]. Improved sources of drinking water included piped water, boreholes or tubewells, protected dug wells, protected springs, rainwater, packaged water. Improved toilet facilities included flush/pour flush toilets connected to piped sewer systems, septic tanks or pit latrines, pit latrines with slabs (including ventilated pit latrines), and composting toilets.

**The wealth quintile was derived from a previously developed abbreviated wealth index using responses to eight questions related to household assets and living conditions, such as ownership of durable goods, housing materials, and access to utilities [12]. Each item was assigned a weight based on its importance in the context of socioeconomic status, which has been calculated reference to standard wealth index in the Nepal Demographic Health Survey 2016 using 42 questions [13]. The simplified wealth index with eight questions has shown to have a kappa statistic of 0.77 to the standard wealth index** [12].

## Statistical Analysis:

- **Lines 125-143:** Provide a justification for using hierarchical logistic spatial regression models and discuss any assumptions made. Explain why Bayesian models were chosen and the advantages they offer in this context.
  - Suggested addition: “Hierarchical logistic spatial regression models were employed to account for the multilevel structure of the data, capturing both individual-level and area-level variations in HEV seroprevalence. Bayesian methods were chosen for their ability to incorporate prior information and provide robust estimates even with complex spatial dependencies. This approach also allows for more accurate uncertainty quantification in the presence of spatial autocorrelation.”

**Author Response**: Thank you for this comment and suggestion. Whilst a Bayesian approach is not required for this analysis, we chose this approach due to its convenient propagation of uncertainty and integrated prediction functionality of INLA. We have now included justification for the use of hierarchical models and use of INLA shown in bold below.

**Line 139-161**: HEV seroprevalence and its 95% confidence interval were estimated based on HEV IgG seroprevalence . We estimated odds ratios (ORs) using univariate logistic regression models to explore associations between demographic factors and HEV seroprevalence. As the study was conducted using bio-banked samples from previously conducted cross-sectional survey with limited HEV risk exposure assessment, no attempt for casual inference was done. We predicted the HEV seroprevalence across Nepal in 2021 on a 5km-by-5km grid by fitting hierarchical logistic spatial regression models to observed seroprevalence data. **Hierarchical logistic spatial regression models were employed to account for the multilevel structure of the data, at both the household-level and community-level.** We obtained grid cell level data on population density and elevation across Nepal from the publicly available sources [14], and travel time to the nearest city from published data [15]. We used publicly available classifications of the three ecological regions [16], and subnational administrative boundaries of Nepal [17] to reflect boundaries as of November 2020. We first assigned each surveyed household to a grid cell and estimated the seroprevalence within each cell containing observations. We then fitted two models to the grid cell level seroprevalence data, firstly a model with spatial covariates for log population density, travel time to the nearest city, and elevation, plus a spatial random effect, and secondly a null model with a spatial random effect only. The models were fitted within a Bayesian framework using integrated nested Laplace approximation (INLA) [18] and used to predict the seroprevalence in the unobserved grid cells. **INLA was chosen due to its convenient propagation of uncertainty, and its computational efficiency over other Markov chain Monte Carlo based approaches to Bayesian model fitting.** We used leave-one-grid-cell-out cross-validation to assess the out-of-sample predictive performance of the two models in terms of mean absolute error, bias, and the strength of the correlation between true observed seroprevalence in left out cells and the predicted seroprevalence. Seroprevalence is summarized with its 95% credible Interval (CrI).

## Results

- **Lines 145-177:** Highlight only key statistical results (e.g., p-values) in the text for important comparisons. Explicitly state the statistical significance of differences in seroprevalence between regions.
  - Suggested addition: “The differences in seroprevalence between the hilly (28.9%, 95% CI: 26.6-31.2) and terai (14.2%, 95% CI: 12.7-15.8) regions were statistically significant (p < 0.001), indicating a higher burden of HEV in the hilly regions.”

**Author Response**: *p*-value has been added where we mention statistical significance.

**Line 169-185**: Of 3,707 survey participants, 3,703 remained in the final analysis after excluding those with invalid laboratory results. Overall, 20.8% (95% CI: 19.54 - 22.16) of the individuals tested positive for anti-HEV IgG. Seroprevalence was consistently low in children, but increased sharply with age among adults sampled. Only 5.8% (95% CI: 1.88 - 9.63) of males and 4.0% (95% CI: 0.16 - 7.76) of females were seropositive in the age group 2-5 years, which contrasts with a much higher 51.6% (95% CI: 44.4 – 58.9) and 42.9% (95% CI: 35.6 – 50.2) in males and females respectively in the age group 60+ years. Overall, male participants exhibited a higher seroprevalence of 23.5% (95% CI: 21.5 – 25.5) compared to females at 18.6% (95% CI: 16.9 – 20.3). This disparity between genders was consistent across age brackets over 20 years old. The three distinct ecological regions of Nepal exhibited variation in seroprevalence. **Seroprevalence was significantly lower (*p* < 0.001), in clusters sampled in the flat Terai region in the south of the country at 14.2% (95% CI: 12.7 – 15.8), compared with the Mountain region in the north of the country at 24.6% (95% CI: 18.8- 30.5), and Hilly region in between at 28.9% (95% CI: 26.6 – 31.2).** There was not a notable difference in seroprevalence between urban (20.6%, 95% CI: 19.0 – 22.2) and rural (21.3%, 95% CI: 19.0-23.6) municipalities (Figure 1). In univariate logistic regression analysis, we found increasing trend of OR as age increases, and male had a statistically higher risk compared to female (OR: 1.39, 95% CI: 1.17-1.65). We didn’t find any statistically significant association between wealth quintile, water source, and toilet facility with HEV IgG seropositivity (Table 1).

### Tables and Figures:

- Ensure all figures are high resolution and clearly labeled. Consider adding more descriptive captions that summarize the key takeaways from each figure/table.
  - Suggested addition for Table 1 caption: “Table 1. Demographic characteristics of study participants and HEV seroprevalence by demographic factors.”
  - Suggested addition for Figure 1 caption: “Figure 1. Hepatitis E Seroprevalence and 95% Confidence Intervals across different demographic and geographic groups.”

**Author Response:** Following suggestions by reviewers on providing more details on sampling methodology, we have inserted Figure 1 to summarize the serosurvey methodology. We have merged Table 1 with Table 2, and same level of details are shown.

Captions have been revised per reviewer’s suggestion as below.

**Figure 1. Overview of Serosurvey Sampling Methodology and Consort Diagram of Study Participants**

**Figure 2. Hepatitis E Seroprevalence and 95% Confidence Intervals across different demographic and geographic groups**

## Discussion

- **Lines 179-247:** Discuss potential policy implications and recommendations for future research more explicitly. Identify specific actions that could be taken based on the study’s findings, such as targeted interventions in high-prevalence areas.
  - Suggested addition: “Our findings suggest the need for targeted interventions in regions with high seroprevalence, particularly the hilly and mountain areas. Public health initiatives should focus on improving water quality and sanitation in these regions. Additionally, the implementation of HEV vaccination programs in high-risk populations, including pregnant women and individuals with chronic liver disease, could significantly reduce the disease burden. Future research should explore the role of zoonotic transmission and the potential benefits of expanding HEV vaccination to broader age groups.”

**Author Response**: We have highlighted key results from the study and their policy implication on the first paragraph of the Discussion. We’ve also added recommendation on targeted public health interventions based on the study findings.

Line 208-213: Seroprevalence varied by region and was generally higher in hilly and mountain regions as compared to terai. Lack of access to clean water may contribute to a higher HEV seroprevalence in these ecological regions as compared to terai region, as Terai is known to have higher coverage of improved water sources than mountain and hilly regions [19]. **Our study findings confirmed that HEV is endemic in Nepal and suggest the need for targeted interventions in regions with high seroprevalence, particularly the hilly and mountain areas. Public health initiatives should focus on improving water quality and sanitation in these regions.**

- **Lines 201-203:** Discuss the limitations in more depth.
  - Current: “The seroprevalence of HEV IgG as a surrogate marker for past infection is likely to be an underestimate as antibodies against HEV are known to wane over time.”
  - Suggested: “The seroprevalence of HEV IgG as a surrogate marker for past infection may underestimate the true infection rate, as antibodies against HEV are known to wane over time. This decline in antibody levels, known as seroreversion, could lead to false negatives, particularly among those infected in the distant past. Additionally, the cross-sectional nature of the study limits the ability to infer causal relationships.”

Author Response: We have revised the paragraph following reviewer’s suggestions. Regarding the comment on the limitation of the study, we have listed limitations of our study all together. We aimed to describe the geospatial distribution of HEV seroprevalence, rather than to infer any causal relationship in our study.

Line 224-227: **The seroprevalence of HEV IgG as a surrogate marker for past infection may underestimate the true infection rate, as antibodies against HEV are known to wane over time.** This decline in antibody levels, known as seroreversion, could lead to false negatives, particularly among those infected in the distant past.

## Conclusion

- **Lines 249-255:** Reinforce the importance of the study’s findings for public health planning and resource allocation. Suggest specific areas for intervention based on the geographic distribution of HEV seropositivity.
  - Suggested addition: “The high seroprevalence in Kathmandu and other urban areas underscores the urgent need for targeted surveillance and intervention programs. Strengthening water, sanitation, and hygiene (WASH) initiatives and considering HEV vaccination for high-risk groups are crucial steps for reducing the burden of HEV in Nepal.”

Author Response: Per reviewer’s suggestion, we have revised the Conclusion paragraph as below.

Line 274-181: The results of this nationally representative serosurvey for HEV in Nepal provide population-based serologic evidence that HEV is endemic in the country, with the highest risk of infection within Kathmandu valley. The considerable geographic variation that we observed in the measured seroprevalence underscores the need for targeted surveillance and intervention programs to make efficient use of limited resources. Targeted strengthening water, sanitation, and hygiene (WASH) initiatives along with potential use of HEV vaccine should be considered in areas with high seroprevalence for high-risk groups, or during an acute outbreak situation, to reduce the burden of HEV in Nepal.

## Overall Assessment

The manuscript is well-structured and provides significant insights into HEV seroprevalence in Nepal. Addressing the suggestions mentioned above will further enhance the manuscript’s clarity, context, and impact. The detailed proofreading notes aim to improve readability and precision.

## Recommendations

1. **Minor Revision:** Address the suggestions and proofreading notes provided above.
2. **Ethical Approval:** Ensure all ethical considerations are clearly documented, particularly regarding bio-banking and informed consent.
3. **Future Research:** Include a brief discussion on future research directions and policy implications based on the findings. Provide more detailed recommendations for policymakers and public health practitioners.

**Author Response:** Taking recommendations, we have addressed suggestions provided above with point-by-point response. We have also provided all the relevant information on ethical approvals obtained for the study on Line 109-112. During the enrollment and consent/assent process of the original serosurvey, participants were consented/assented separately for long term storage and additional testing infectious etiologies including HEV.

Based on the study findings, we have added policy implications on the first paragraph of the Discussion section (Line 210-214), and Conclusion section (Line 276-283). As our study was unable to differentiate HEV infection by genotypes, we recommended future studies to address this gap (Line 266-268).

## Specific Areas for Improvement

1. **Introduction:** Provide more background on HEV epidemiology in Nepal and previous studies to set the stage for the current research.

**Author Response:** On the second paragraph of the Introduction, we have provided previous reports on HEV epidemiology in Nepal. We highlighted that despite several studies reporting endemicity of HEV in Nepal, nationally representative disease burden data on HEV are lacking in order to set the stage for the current research (Line 68-80).

2. **Methods:** Clarify sampling criteria, provide more detail on laboratory quality control, and justify the choice of statistical models.

**Author Response:** We have elaborated with more details on how Nepal is divided administratively and ecologically. Then we added more details on sampling method per reviewer suggestion. To better clarify the process, we have inserted Figure 1 to briefly summarize sampling method and consort diagram of study participants (Line 89-101).

We have provided rationale for statistical models chosen for this study (Line 140-162) under the Methods – Statistical Analysis section.

3. **Results:** Highlight key statistical findings more clearly and discuss observed patterns in greater detail.

**Author Response:** Where applicable, we have added p-value in the main text or used 95% CIs. We aimed to describe HEV seroprevalence by age, ecological regions, and municipality types. When showing geospatial distribution, we primarily used ecological regions as a layer for the mapping. An alternative geospatial map showing the boundaries of seven provinces has been added as Supplementary Figure 1 to shown seroprevalence patterns in multiple ways.

4. **Discussion:** Expand on policy implications and future research directions. Address limitations in more depth.

**Author Response:** Based on the study findings, we have added policy implications on the first paragraph of the Discussion section (Line 210-214), and Conclusion section (Line 276-283). As our study was unable to differentiate HEV infection by genotypes, we recommended future studies to address this gap (Line 266-268).

5. **Figures and Tables:** Ensure high-resolution images and add more descriptive captions. Consider including additional visualizations to illustrate regional differences.

**Author Response:** Captions for the tables and figures have been revised to provide more description and sources. An alternative geospatial map showing the boundaries of seven provinces has been added as Supplementary Figure 1 to shown seroprevalence patterns in multiple ways.

6. **References:** Update your references to the most recent literature, the oldest should be five years ago and not beyond that, also ensure consistency in formatting. Include more recent studies.

**Author Response:** All the references have been checked and updated. There are few old references used in the study due to limited publications on HEV in Nepal. We have used references on HEV epidemiology in Nepal that were reported more than five years ago only when necessary (i.e., history of HEV outbreak in Nepal).

Thank you.

Chulwoo Rhee, MD MSc

Date: 10 OCT 2024

Email: [rhee275@gmail.com](mailto:rhee275@gmail.com)
